# A Prospective Study of Inhaled Therapy Prescribing, Pressurised Metered-Dose Inhaler (pMDI) Technique Assessment, and Inhaler Concordance Monitoring Among Hospitalised Adult Patients With Airways Disease

**DOI:** 10.7759/cureus.97583

**Published:** 2025-11-23

**Authors:** Luanne Lai, Bethany Cockerill, Emma Lunn, Reem Abdelgardir, Yong Xuan Teoh, George Antunes

**Affiliations:** 1 Urology, Wirral University Teaching Hospital NHS Foundation Trust, Wirral, GBR; 2 Respiratory Medicine, South Tees Hospitals NHS Foundation Trust, Middlesbrough, GBR; 3 General Medicine, North Tees and Hartlepool NHS Foundation Trust, Stockton-on-Tees, GBR; 4 General Medicine, South Tees Hospitals NHS Foundation Trust, Middlesbrough, GBR

**Keywords:** asthma, copd: chronic obstructive pulmonary disease, inhaler technique, inhaler therapy, test of adherence to inhalers questionnaire

## Abstract

Introduction

Prescribing of inhaled therapy, pressurised metered-dose inhaler (pMDI) technique at hospitalisation, and therapy concordance pre-admission remains inadequate. A study by Asthma UK (2010-2013) found over 125,000 asthma prescribing errors and that one-third of patients had ineffectual inhaler technique.

Aims and objectives

This study aims to identify the adequacy of inhaled therapy prescriptions and assess pMDI technique and pre-admission inhaler concordance among patients with airways disease using the pMDI technique checklist and the Test of Adherence (TAI) questionnaire.

Methods

A two-centre prospective study was performed over three months in 2022 at two secondary-care hospitals in the UK. Data were obtained from prescriptions, including availability of inhalers at 24 and 72 hours and the number of devices. A pMDI technique checklist and assessment of concordance using the TAI questionnaire were performed by trained practitioners.

Results

Seventy patients were recruited (median age = 73; chronic obstructive pulmonary disease (COPD): n=50, asthma: n=20). Inhaled therapy was prescribed correctly in 80% (n=56); 54% (n=38) and 70% (n=49) had their inhalers supplied within 24 and 72 hours of hospital admission, respectively. Sixty-nine percent (n=48) used two or more different devices on admission. Fifty-seven percent (n=39) had ≥1 critical error with pMDI inhaler use. Seventy-one percent (n=48) of patients were graded as “good adherence” on the TAI questionnaire.

Conclusions

Inhaled therapy was well prescribed, but availability of inhaled devices within 48 hours of admission could be improved. Assessment of pMDI inhaler technique during hospitalisation is recommended. Initiation of combination inhalers for patients using two or more different devices may improve concordance. The TAI was used in an acute setting for the first time, but a larger study is needed to evaluate its full potential during hospitalisation.

## Introduction

Prescriptions of inhaled therapies, along with ineffectual inhaler technique and non-concordance, can have a significant negative impact on the management of airway diseases. A study by Asthma UK (2010-2013), looking at data from the Optimum Patient Care Research Database, estimated that over 120,000 patients across the United Kingdom had unsafe prescribing practices, which increased their risk of an exacerbation [1]. A National Institute for Health and Care Excellence (NICE) literature review [2] also found that 30-70% of patients had variances in their medication pre-hospital and during admissions.

Inhaler concordance is important to achieve positive patient outcomes and reduce the burden on healthcare resources [3]. The number of inhalation devices and multiple dissimilar inhalation manoeuvres may lead to reduced concordance, this becomes relevant as 60-70% of inhalers used in the UK are pressurised metered-dose inhalers (pMDIs) [4]. A ‘real-world’ study [5] observed a higher rate of concordance and persistence after 12 months in individuals who used a single inhaler compared to multi-inhaler therapy, while a large cohort study [6] demonstrated that patients with similar device therapy had lower exacerbation rates compared to those who used mixed devices.

Poor inhaler technique has been shown to contribute to suboptimal patient outcomes. A well-conducted study estimated that up to 85% of patients do not use their inhalers correctly because of the complexity required to ensure a correct inhalation manoeuvre [7]. This is more common in the older population, where erroneous inhaler technique can serve as a predictive tool for assessing individual risk of exacerbations [8].

The Test of Adherence to Inhalers (TAI) questionnaire is a useful tool to assess and monitor inhaler concordance in a clinic or outpatient setting and has been validated by several studies [9,10].

The study aimed to identify the quality of inhaled therapy prescriptions at hospital admission, the number of different devices used by each individual, the adequacy of pMDI technique using the MDI inhaler checklist by van der Palen J et al. [11], and pre-admission inhaler concordance using the TAI questionnaire.

## Materials and methods

Patients were recruited prospectively from the Acute and Respiratory Medicine services at two secondary care hospitals in the Northeast of England (n=70).

The principal inclusion criteria were a pre-existing diagnosis of chronic obstructive pulmonary disease (COPD) or asthma at hospital admission, use of a pMDI inhaler as rescue medication, age above 18 years, and the ability to consent to undertake and complete the relevant assessments and questionnaires. Prescribing information, obtained from paper and online prescriptions, included the quality of inhaler prescriptions at 24, 48, and 72 hours following hospitalisation (rounded up to the next full day) based on NICE guidance for prescription writing [12], the type and number of devices utilised by each patient, and the availability of inhalers at the patient's bedside. Patients not utilising pMDIs were excluded from the study, this consisted of a very small number, as most patients overwhelmingly used pMDIs (n=8). Assessments and questionnaires were performed by trained medical and nursing teams based in the respective wards.

**Figure 1:**
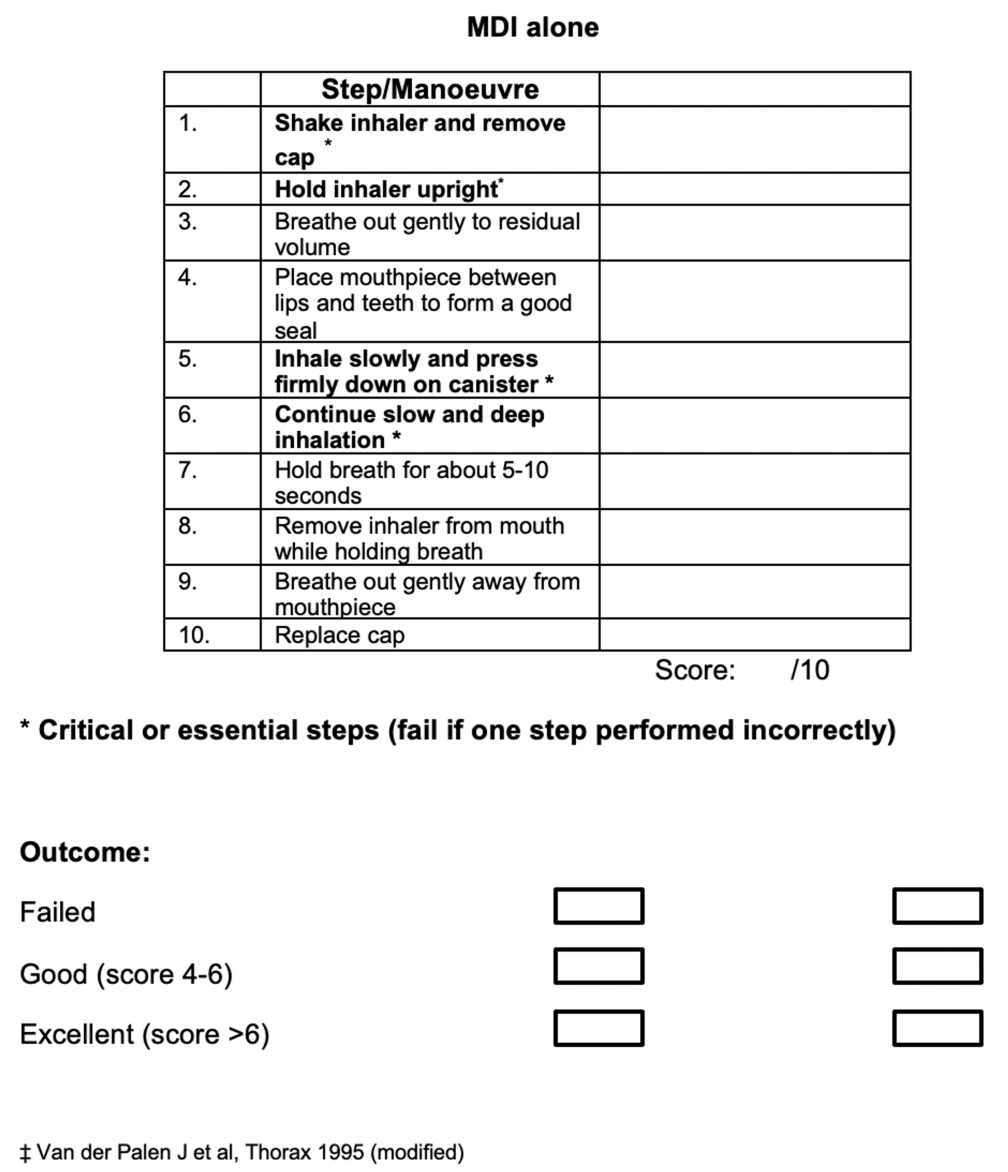
pMDI inhaler technique checklist. Adapted from van der Palen J et al. [11]. Permission to reproduce the material was obtained from the original publisher.

Patients were formally consented prior to performing a standard 11-point pMDI inhaler technique checklist (Figure 1) and the TAI questionnaire. The pMDI inhaler technique checklist consisted of predefined and validated critical errors, and patients failed the test if one or more critical errors were identified. Permission to use the English format of the TAI questionnaire was obtained, and scores were recorded according to the original authors’ instructions or guidance. Scores for the 11-point pMDI inhaler technique checklist and the TAI questionnaire were recorded for each patient.

Statistical analysis

Data analysis was performed using SPSS software. A Shapiro-Wilk test was used to determine whether the dataset was parametric. Further analysis was conducted to describe the data using means, medians, ranges, and SD, and Fisher’s exact test was utilised to determine the significance of findings.

Ethical considerations

This study involved human participants. The UKRI/MRC decision tool was used to determine whether NHS REC review was required, and it confirmed that NHS REC approval was not needed for this study.

## Results

A total of 70 patients were recruited over a three-month period, of which 71.4% (n=50) had a diagnosis of COPD and 28.6% (n=20) had asthma. The mean age of patients was 69.5 years (SD ±16.2). The percentage of male patients was 38.6% (n=27) and female patients was 61.4% (n=43).

Overall, 75.7% (n=53) of patients had an inpatient duration of 1-3 days, 14.3% (n=10) stayed for 4-7 days, 5.7% (n=4) stayed for 8-14 days, and 4.3% (n=3) stayed longer than 2 weeks. Inhaled therapy prescriptions were adequately prescribed for 80% (n=56) of patients on admission. The most frequently missed feature was the start date of inhaled therapy.

**Figure 2:**
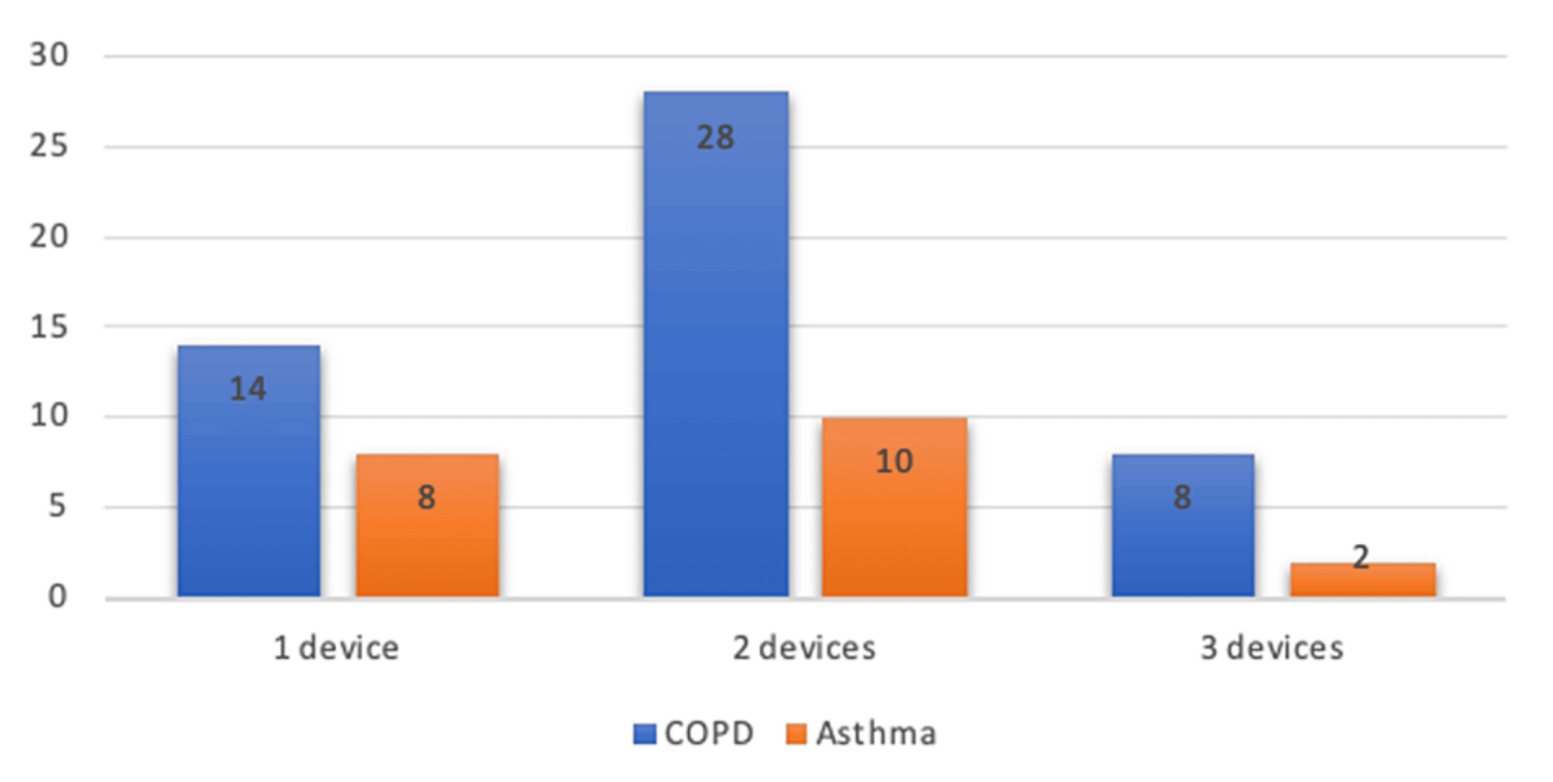
Number of inhalers used by patients. COPD: Chronic obstructive pulmonary disease.

A total of 68.6% (n=48) of patients were admitted on ≥2 inhalers (Figure 2), and there was no significant difference between asthma patients (60% (n=12)) and COPD patients (72% (n=36)). All asthma patients were on inhaled corticosteroid therapy. Inhaled therapy was available at the bedside within 24 hours in 54% (n=38) of patients and within 72 hours in 70% (n=49) of patients.

All but one patient consented to the MDI inhaler technique assessment. Of these, 43% (n=30) demonstrated excellent inhaler technique, i.e., no critical errors. The most frequently missed step in the MDI inhaler technique checklist was “continue slow and deep breaths,” while the most common critical error was failing to “breathe out gently to residual volume” prior to using the pMDI inhaler. A total of 15.9% (n=11) of patients were able to score full marks in the MDI inhaler technique assessment.

Sixty-eight patients completed the TAI questionnaire. Overall, 70.6% (n=48) scored “good adherence” (≥50 points), and 7.4% (n=5) scored “intermediate adherence” (46-59 points). A total of 22.1% (n=15) had scores consistent with “poor adherence” (≤45 points). Among patients who scored “good adherence,” 75% (n=36) had COPD and 25% (n=12) had asthma, which was not statistically significant (p = 0.432).

## Discussion

The study’s main aims were to assess aspects of prescribing inhaled therapy at, and shortly after, hospitalisation for an exacerbation. It also provided an opportunity to evaluate pMDI inhaler technique and monitor concordance with inhaler therapy using a validated questionnaire.

Our findings showed that prescribing practices at this time point were good. However, the availability of inhalers at a patient’s bedside within 72 hours of admission was modest. This aspect has not been studied formally, to the best of our knowledge, but is probably a critical factor in promoting and maintaining inhaler concordance.

We found that most patients (n=48) had ≥2 inhalers on admission in both asthma and COPD groups. This is significant for a subset of patients who could potentially be switched to single-inhaler or combination inhaler therapy. A recent study by Vanoverschelde A et al. [13] demonstrated that the strongest determinant of poor inhaler technique was the use of multiple inhalers and inhalation manoeuvres. We believe this is an opportune time point to reduce the number of inhaler devices, simplify therapy, and improve patient outcomes significantly [14,15].

Switching to combination inhalers was undertaken in several patients during hospitalisation. A patient-centred approach was used to change inhalers while addressing other aspects of management, e.g., smoking cessation and pulmonary rehabilitation. Switching inhaled therapy may have a beneficial impact on disease control, the selection of environmentally friendly inhalers, and cost reduction, but requires shared decision-making [16,17]. Although hospitalisation appears to be an appropriate opportunity to switch inhaled therapy by trained healthcare professionals, with onward monitoring in primary care, a larger study is required to support this intervention [2,18].

pMDIs are responsible for 0.03% of annual global greenhouse gas emissions and account for 3% of the NHS’s total prescriptions [19]. Switching to an environmentally friendly pMDI or dry powder inhaler can significantly reduce CO₂ emission burden while remaining cost neutral [20,21]. However, this is easier said than done, as switching away from pMDIs may not always be feasible depending on the individual’s inhaler regimen. For example, based on the Global Initiative for Asthma (GINA) guidelines, depending on the patient’s treatment track, they may still utilise pMDIs as a reliever inhaler (e.g., short-acting beta-agonists (SABA) in Track 2), which needs to be balanced against environmental considerations [22]. This also applies to COPD where, according to the Global Initiative for Chronic Obstructive Lung Disease (GOLD) guidelines, patients are generally given pMDIs as a reliever inhaler alongside other maintenance inhalers. The choice of inhaler also depends on the patient’s ability to take long, deep breaths for metered-dose inhalers (MDIs) [23]. Current evidence from both GINA and GOLD still heavily supports the use of pMDIs for reliever treatment. Hence, although it is prudent to consider the environmental impact of pMDI inhalers, any inhaler changes should be pragmatic and primarily aimed at benefitting the overall health of the patient.

Poor inhaler technique is associated with a reduction in disease control, an increased number of exacerbations, and increased healthcare spending [24]. Incorrect inhaler technique, defined as exhibiting one or more critical errors, was demonstrated in 57% of patients, which is consistent with published data [25]. Hospital admission provides an excellent opportunity for health professionals and patients to review inhaler technique, with continuing assessment of inhaler technique in primary care [26].

Concordance with inhaler use was evaluated using the validated TAI questionnaire, and 70% of patients scored “good adherence.” The TAI questionnaire has been extensively validated in a variety of settings for both COPD and asthma patients [9,10]. It remains a useful tool despite the availability of other methods to assess and monitor concordance with inhaled therapy. One limitation of the TAI questionnaire is that it does not always account for the clinical and sociodemographic characteristics of patients. This was reflected in our experience, where older patients were often confused by the wording and repetitiveness of certain questions. Our study has shown the feasibility of utilising the TAI questionnaire in an acute setting, which could encourage further studies to validate the questionnaire in larger populations.

The main limitation of the study was its size, and specifically the disparity in the number of patients with COPD versus asthma. This precluded the assessment of meaningful differences between the groups in relation to several objectives. Another limitation to consider is the age range of our patients, one of the hospitals where we collected data catered predominantly to an ageing population, which could contribute to greater use of multiple inhalers and poorer pMDI technique.

## Conclusions

This study provided insight into current prescribing practices of pMDI inhalers by healthcare professionals at our trust, the assessment of inhaler technique, and the measurement of concordance with inhaled therapy among patients with airways disease at, and shortly after, admission to hospital. Further improvements could be made in several areas: medicines reconciliation at the bedside, routine bedside pMDI technique checks, and structured handovers for the ongoing management of these patients in the community could have a direct impact on their care. Other potential areas of interest for future studies include evaluating the use of combination inhaler therapy and promoting interventions that could improve overall concordance and disease control among this group of patients.
